# *Acinetobacter baumannii* Secretes a Bioactive Lipid That Triggers Inflammatory Signaling and Cell Death

**DOI:** 10.3389/fmicb.2022.870101

**Published:** 2022-05-09

**Authors:** Varnesh Tiku, Chun Kew, Eric M. Kofoed, Yutian Peng, Ivan Dikic, Man-Wah Tan

**Affiliations:** ^1^Department of Infectious Diseases, Genentech, South San Francisco, CA, United States; ^2^Faculty of Medicine, Institute of Biochemistry II, Goethe University Frankfurt, Frankfurt, Germany; ^3^Buchmann Institute for Molecular Life Sciences, Goethe University Frankfurt, Frankfurt, Germany; ^4^Max Planck Institute of Biophysics, Frankfurt, Germany

**Keywords:** *Acinetobacter baumannii*, virulence, cell death, inflammatory signaling, inflammasome, NF-κB activation

## Abstract

*Acinetobacter baumannii* is a highly pathogenic Gram-negative bacterium that causes severe infections with very high fatality rates. *A. baumannii* infection triggers innate as well as adaptive immunity, however, our understanding of the inflammatory factors secreted by *A. baumannii* that alarm the immune system remains limited. In this study, we report that the lab adapted and clinical strains of *A. baumannii* secrete an inflammatory bioactive factor which activates TLR2, leading to canonical IRAK4-dependent NF-κB signaling and production of pro-inflammatory cytokines interleukin (IL)-6 and IL-8 and activation of the inflammasome pathway causing pyroptotic cell death. Biochemical fractionation of the *A. baumannii* culture filtrate revealed the hydrophobic nature of the inflammatory factor. Concordantly, lipase treatment of the culture filtrate or TLR2 inhibition in macrophages abrogated NF-κB activation and cell death induction. Culture filtrates from the LPS- and lipoprotein-deficient *A. baumannii* mutants retain immuno-stimulatory properties suggesting that a lipid other than these known stimulatory molecules can trigger inflammation during *A. baumannii* infection. Our results reveal that *A. baumannii* secretes a previously unappreciated inflammatory bioactive lipid that activates multiple pro-inflammatory signaling pathways and induces cell death in human and murine macrophages.

## Introduction

*Acinetobacter baumannii* is a Gram-negative bacterium that causes severe infections in humans that can lead to multifaceted clinical manifestations including pneumonia, meningitis and septicemia often with very high mortality rates (Wong et al., 2017). One of the major reasons behind the emergence of *A. baumannii* as a threat to public health is its extensive resistance to currently available antibiotics. *A. baumannii* is known to have higher expression levels of efflux pumps and ß-lactamase enzymes that degrade antibiotics which eventually lead to enhanced antibiotic resistance (Coyne et al., 2011; Raible et al., 2017).

Upon infection, *A. baumannii* induces a potent immune response which is mainly driven by pathogen recognition receptors (PRRs) in immune and epithelial cells. The major PRR that drives the production of inflammatory cytokines during *A. baumannii* infection is the Toll-Like Receptor 4 (TLR4) (Chen, 2020). Macrophages and dendritic cells exhibit strong induction of TLR4, driven primarily by lipopolysaccharide (LPS) during *A. baumannii* infection, which leads to the activation of the transcription factor NF-κB and downstream production of cytokines including interleukin (IL)-6, IL-12 and tumor necrosis factor (TNF)- α, (Kim et al., 2013; Erridge et al., 2007). TLR4 knock out mice display a significantly delayed inflammatory response that eventually leads to higher survival and reduced mortality upon *A. baumannii* infection (Knapp et al., 2006; Lin et al., 2012). TLR2 is another important plasma membrane associated PRR which is known to serve pivotal functions in mounting immune responses against bacterial infections. However, the role of TLR2 during *A. baumannii* infection is less well-understood (Chen, 2020). *A. baumannii* infection is also known to induce the inflammasome pathway which induces pyroptosis and the secretion of inflammatory cytokines IL-1ß and IL-18 (Dikshit et al., 2018). The immune responses reported upon *A. baumannii* infection have been associated majorly with well characterized pathogen associated molecular patterns (PAMPs) such as LPS. And in order to evade immune recognition and to gain resistance against antibiotics that target LPS, *A. baumannii* can readily rid itself of LPS (Moffatt et al., 2010; Cai et al., 2012). However, it remains to be established if *A. baumannii* possesses any alternative factors other than LPS that serve as PAMPs to alarm the immune system. It is also not known if *A. baumannii* secretes any immunomodulatory factors that can trigger immune responses in host cells. Since hyperinflammation during *A. baumannii* infections are primary drivers of lethality in the host, it is crucial to examine if *A. baumannii* possesses additional factors that might be involved in triggering immune responses (Bruhn et al., 2015; Wong et al., 2017).

In this study, we analyzed the secreted factors of *A. baumannii* for their capacity to stimulate an inflammatory response. We observed that the culture filtrates from the lab-adapted and the clinical isolates of *A. baumannii* potently induced canonical NF-κB signaling and the inflammasome pathway leading to cell death in macrophages. LPS is shed by *A. baumannii* which can activate NF-κB (Lin et al., 2012), however, we observed that the culture filtrate from the LPS deficient Δ*lpxA* mutant still induced inflammation. Further characterization ruled out the possibility of DNA, RNA, proteins, lipoproteins and cyclic nucleotides as the secreted activating factor. Biochemical analysis revealed the hydrophobic nature of the inflammatory factor in the culture filtrate. In accord with this finding, lipase treatment of the culture filtrate from *A. baumannii* abolished the activation of NF-κB and inflammasome pathways resulting in reduced pro-inflammatory cytokine production and reduced pyroptotic cell death. Our results suggest that *A. baumannii* secretes a novel bioactive lipid detected by TLR2 that acts as a potent inflammatory signal in macrophages and either lipase treatment of *A. baumannii* culture filtrate or TLR2 inhibition in macrophages blocks the downstream inflammatory signaling.

## Results

### *Acinetobacter baumannii* Secretes Pro-inflammatory Factors

**FIGURE 1:**
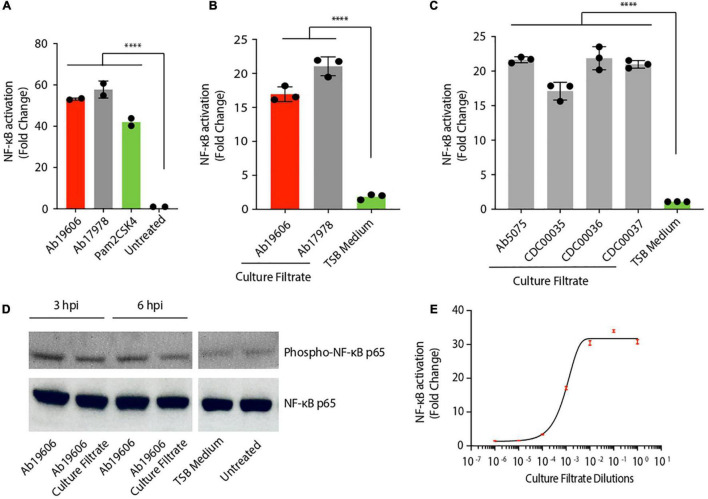
*Acinetobacter baumannii* secretes pro-inflammatory factors. **(A)** THP1-XBlue reporter cells infected with *A. baumannii* (Ab19606 and Ab17978) at MOI 10. Levels of secreted embryonic alkaline phosphatase (SEAP) were assessed after 24 h of infection. The experiments were done in triplicates. Error bars represent standard deviation. One-way ANOVA with Tukey’s multiple comparisons test. **(B)** THP1-XBlue reporter cells treated with culture filtrates from *A. baumannii* (Ab19606 and Ab17978). TSB medium was used to grow the bacterial cultures and fresh TSB medium treatment served as the negative control. Levels of SEAP were assessed after 24 h from the beginning of the treatment. The experiments were done in triplicates. Error bars represent standard deviation. One-way ANOVA with Tukey’s multiple comparisons test. **(C)** THP1-XBlue reporter cells treated with culture filtrates from the indicated clinical isolates of *A. baumannii*. TSB medium was used to grow the bacterial cultures and fresh TSB medium treatment served as the negative control. Levels of SEAP were assessed after 24 h from the beginning of the treatment. The experiments were done in triplicates. Error bars represent standard deviation. One-way ANOVA with Tukey’s multiple comparisons test. **(D)** Western blot to check the phosphorylation of NF-κB p65 in RAW264.7 macrophages after the indicated infection (at MOI 10) or culture filtrate treatment. **(E)** THP1-XBlue reporter cells treated with different dilutions of the culture filtrate from *A. baumannii* (Ab19606). 10-fold serial dilutions of the culture filtrate were done in fresh TSB medium. Levels of SEAP were assessed after 24 h from the beginning of the treatment. The experiments were done in triplicates. Error bars represent standard deviation. All the experiments were performed three times independently. *P < 0.05, **P < 0.01, ***P < 0.001, ****P < 0.0001.

It is well known that *A. baumannii* infection activates inflammatory signaling pathways (Harding et al., 2017; Chen, 2020). Similar to published results, THP1 human monocytic cells stably expressing NF-κB inducible secreted embryonic alkaline phosphatase (SEAP) reporter gene, exhibited NF-κB activation upon infection with two different strains of *A. baumannii*, Ab19606 and Ab17978 (Figure 1A) (Kim et al., 2013). Pam2CSK4, which is a known activator of NF-κB, validated our experimental system (Figure 1A). To test if the immunomodulatory factors are secreted by *A. baumannii*, bacteria-free culture filtrates from overnight *A. baumannii* cultures were tested and shown to also activate NF-κB (Figure 1B). We verified the absence of bacteria in the culture filtrates by standard colony forming unit (CFU) plating assay (Supplementary Figure 1A). Treatment of the reporter cells with culture filtrate from clinical isolates of *A. baumannii* also activated NF-κB, highlighting the relevance of these findings in clinically important strains (Figure 1C). To further confirm the NF-κB activation results we treated murine RAW264.7 macrophages with *A. baumannii* or its culture filtrate and assessed the phosphorylation of NF-κB by western blotting. Similar to our reporter assay results (Figures 1A,B), both *A. baumannii* infection and the culture filtrate treatment enhanced the phosphorylation of NF-κB suggesting its activation (Figure 1D). Treatment of the THP1 reporter cells with different dilutions of the culture filtrate yielded a typical dose-response curve revealing a concentration dependence of the activating factor in its capacity to trigger NF-κB (Figure 1E). Taken together, these results suggest that both clinical and lab-adapted strains of *A. baumannii* secrete potent inflammatory molecules that activate the major immunomodulatory transcription factor NF-κB in human and murine macrophages.

### *Acinetobacter baumannii* Secretes Factors That Activate Inflammatory Cytokines and Pyroptosis

**FIGURE 2:**
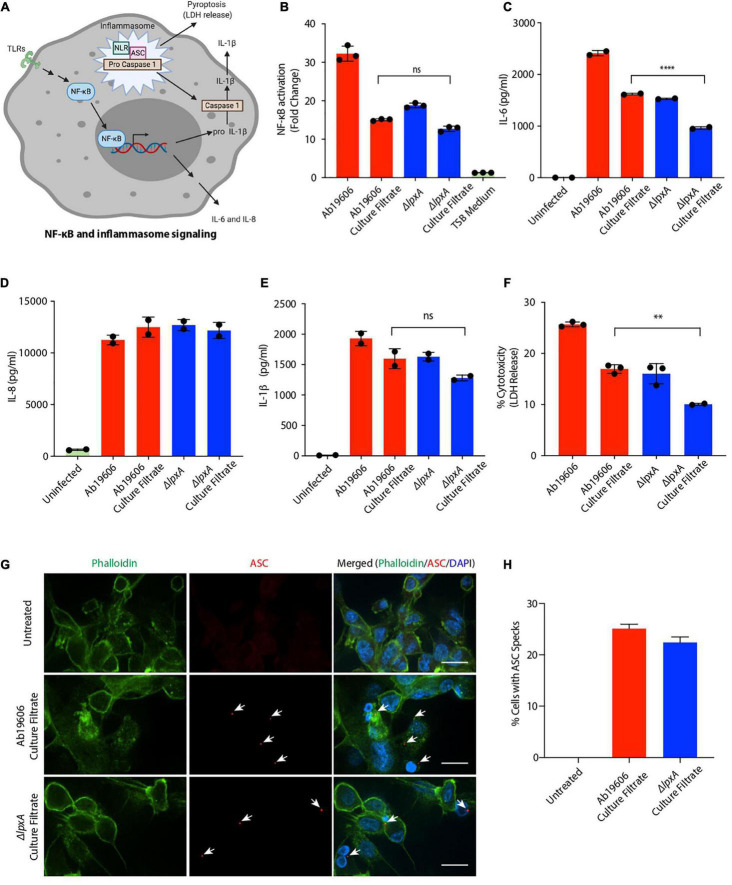
*Acinetobacter baumannii* secretes factors that activate inflammatory signaling and pyroptosis. **(A)** Schematic depicting the NF-κB and the inflammasome signaling pathways; TLRs – Toll-like receptors, NLR – Nod-like receptor (created with BioRender.com). **(B)** THP1-XBlue reporter cells were infected with wildtype *A. baumannii* (Ab19606) and Δ*lpxA* at MOI 10 or treated with the respective culture filtrates. Levels of SEAP were assessed after 24 h of infection or culture filtrate treatment. The experiments were done in triplicates. Error bars represent standard deviation. One-way ANOVA with Tukey’s multiple comparisons test. **(C–E)** THP1 macrophages were infected with wildtype *A. baumannii* (Ab19606) and Δ*lpxA* at MOI 10 or treated with the respective culture filtrates. Levels of IL-6, IL-8 and IL-1ß in the cell culture supernatant after 24 h of infection or culture filtrate treatment were assessed by ELISA. The experiments were done in triplicates. Error bars represent standard deviation. One-way ANOVA with Tukey’s multiple comparisons test ns – not significant. **(F)** THP1 macrophages were infected with wildtype *A. baumannii* (Ab19606) and Δ*lpxA* at MOI 10 or treated with the respective culture filtrates. LDH levels in the cell culture supernatant were assessed after 24 h of infection or culture filtrate treatment. The experiments were done in triplicates. Error bars represent standard deviation. One-way ANOVA with Tukey’s multiple comparisons test. **(G)** THP1 macrophages were treated with the indicated culture filtrates for 6 h. The cells were then fixed and immunofluorescence was performed to detect ASC specks using an anti-ASC antibody. Arrowheads indicate ASC specks. The scale bars represent 20 μM. **(H)** The graph represents % cells with ASC specks relative to the total number of cells counted in the indicated treatments. All the experiments were performed three times independently. *P < 0.05, **P < 0.01, ***P < 0.001, ****P < 0.0001.

Bacterial PAMPs trigger multiple signaling pathways in host cells including the NF-κB and the inflammasome pathways depicted in Figure 2A. Lipopolysaccharide is the most common PAMP associated with Gram-negative bacteria that triggers inflammation. We next investigated whether LPS was the inflammatory factor in the *A. baumannii* culture filtrate that stimulated NF-κB. We generated the LPS deficient strain by replacing the *lpxA* gene with a kanamycin cassette using genetic recombineering (Supplementary Figure 1B) (Bojkovic et al., 2016). *lpxA* is required for the biosynthesis of the Lipid A moiety of LPS and its inactivation results in complete loss LPS in *A. baumannii* rendering it resistant to the antibiotic colistin which targets LPS (Moffatt et al., 2010). Inactivation of *lpxA* resulted in colistin resistance suggesting the loss of LPS production as previously described (Supplementary Table 1) (Moffatt et al., 2010). NF-κB reporter cells infected with Δ*lpxA* displayed reduced NF-κB activation compared to wildtype *A. baumannii* infection (Figure 2B). However, treatment of the reporter cells with bacteria-free culture filtrates from Δ*lpxA* and wildtype *A. baumannii* exhibited comparable activation of NF-κB (Figure 2B). These data suggest the presence of a pro-inflammatory factor other than LPS in the culture filtrate from *A. baumannii* that can activate NF-κB. We also analyzed culture filtrates from lipoprotein mutants and observed no difference between the culture filtrates of the wildtype and the lipoprotein mutants in their ability to activate NF-κB ruling out the possibility of lipoproteins as being the inflammatory signal in *A. baumannii* culture filtrates (Supplementary Figures 1C,D). We next examined if *A. baumannii* culture filtrate treatment induces the release of pro-inflammatory cytokines. Consistent with NF-κB activation, treatment of THP1 macrophages with the culture filtrate from wildtype and Δ*lpxA A. baumannii* led to enhanced secretion of the pro-inflammatory cytokines IL-6 and IL-8 (Figures 2C,D). While the secretion of IL-6 was partially dependent on the presence of LPS, IL-8 secretion did not display any LPS dependent effects (Figures 2C,D). Interestingly we also observed enhanced secretion of IL-1ß in THP1 macrophages treated with the culture filtrate from wildtype and Δ*lpxA A. baumannii* (Figure 2E). Since IL-1ß is released upon pyroptotic cell death (Figure 2A) (He et al., 2016; Man et al., 2017), we next asked if *A. baumannii* culture filtrate treatment also induces cell death. THP1 macrophages treated with the culture filtrate from wildtype and Δ*lpxA A. baumannii* induced cell death (Figure 2F). LPS was seen to play only a partial role in mediating cell death (Figure 2F). Pyroptotic cell death is mediated by the inflammasome pathway which requires the recruitment of the adaptor protein apoptosis associated speck-like protein containing a CARD (ASC) for the downstream activation of Caspase 1 (Guo et al., 2015) (Figure 2A). Therefore, we examined ASC speck formation which is considered a hallmark of the inflammasome activation (Guo et al., 2015). THP1 cells treated with the culture filtrate from wildtype and Δ*lpxA A. baumannii* displayed clearly visible ASC specks indicative of the activation of the inflammasome pathway (Figures 2G,H). Taken together these results suggest that *A. baumannii* secretes a potent inflammatory molecule, other than the previously established LPS, that induces different inflammatory pathways in macrophages including NF-κB activation and the inflammasome pathway.

### Characterization of Inflammatory Factors in *Acinetobacter baumannii* Culture Filtrate

**FIGURE 3:**
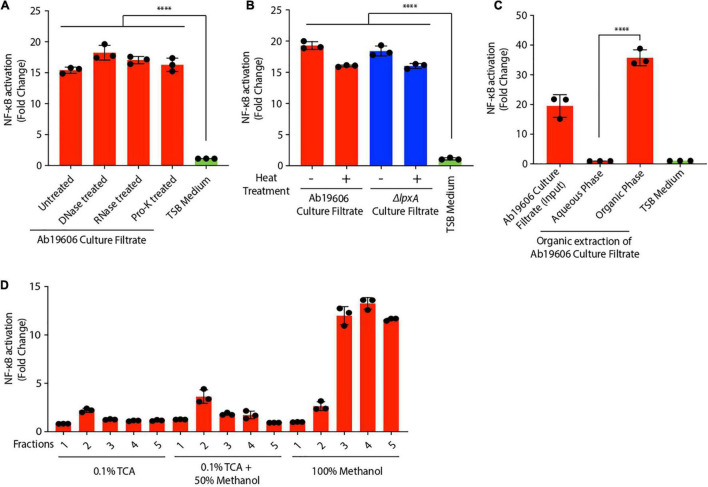
Characterization of inflammatory factors in *Acinetobacter baumannii* culture filtrate. **(A,B)** Culture filtrate from wildtype *A. baumannii* (Ab19606) was treated with DNase, RNase, proteinase K (Pro-K) or heat treated. THP1-XBlue reporter cells were exposed to untreated and the treated culture filtrate. Levels of SEAP were assessed after 24 h of culture filtrate treatment. The experiments were done in triplicates. Error bars represent standard deviation. One-way ANOVA with Tukey’s multiple comparisons test. **(C)** Organic extraction was performed on the culture filtrate from wildtype *A. baumannii* (Ab19606). THP1-XBlue reporter cells were treated with the Aqueous and organic phases. TSB medium was used to grow the bacterial cultures and fresh TSB medium treatment served as the negative control. Levels of SEAP were assessed after 24 h from the beginning of the treatment. The experiments were done in triplicates. Error bars represent standard deviation. One-way ANOVA with Tukey’s multiple comparisons test. **(D)** Biochemical fractionation was performed on the culture filtrate from wildtype *A. baumannii* (Ab19606). THP1-XBlue reporter cells were treated with the indicated fractions. Levels of SEAP were assessed after 24 h from the beginning of the treatment. The experiments were done in triplicates. Error bars represent standard deviation. One-way ANOVA with Tukey’s multiple comparisons test. All the experiments were performed three times independently. *P < 0.05, **P < 0.01, ***P < 0.001, ****P < 0.0001.

In order to ascertain the molecular identity of the inflammatory factor, we treated the culture filtrate from wildtype *A. baumannii* with DNase, RNase and proteinase K, to remove the known immune activators – DNA, RNA and proteins (Klinman et al., 1996; Rahman and McFadden, 2011; Eigenbrod and Dalpke, 2015). None of these treatments abolished NF-κB activation induced by the culture filtrate (Figure 3A). Moreover, heat treatment of the culture filtrate only slightly reduced its ability to activate NF-κB suggesting that the major contributing factor in *A. baumannii* culture filtrate that triggers NF-κB is not heat-labile (Figure 3B). We also treated wildtype *A. baumannii* culture filtrate with phosphodiesterase (PDE) enzyme to neutralize bacterial cyclic dinucleotides which are known to activate immune responses (Woodward et al., 2010; Danilchanka and Mekalanos, 2013). However, PDE treatment of the culture filtrate did not abrogate NF-κB activation (Supplementary Figure 2A). The functionality of the PDE treatment was verified by assessing ATP levels with or without PDE treatment (Supplementary Figure 2B). Bacterial outer membrane vesicles (OMVs), which are immune-stimulatory, were extracted from *A. baumannii* culture filtrate, however, the OMV deficient culture filtrate still activated NF-κB ruling out OMVs as being the inflammatory factor (Kaparakis-Liaskos and Ferrero, 2015; Tiku et al., 2021) (Supplementary Figure 2C). *A. baumannii* OmpA is an important virulence factor that induces cytotoxicity and immune activation in host cells (García-Patiño et al., 2017; Tiku et al., 2021). However, the culture filtrate from Δ*ompA* only slightly reduced NF-κB activation in the reporter cells suggesting that OmpA is not the major trigger for NF-κB stimulation (Supplementary Figure 2D).

To further characterize the NF-κB activating factor secreted by *A. baumannii*, we utilized biochemical approaches. Organic phenol-chloroform extraction performed on *A. baumannii* culture filtrate revealed that the NF-κB activating factor was present in the organic fraction and not in the aqueous fraction (Figure 3C). Concordantly reverse-phase biochemical fractionation of the culture filtrate showed that the NF-κB activating factor was eluted in the 100% methanol fraction suggesting that the factor is hydrophobic (Figure 3D). Altogether, these data suggest that *A. baumannii* secretes a hydrophobic NF-κB stimulating factor that is not LPS or other known immune-modulators.

### *Acinetobacter baumannii* Secretes a Bioactive Lipid That Triggers Immune Signaling

**FIGURE 4:**
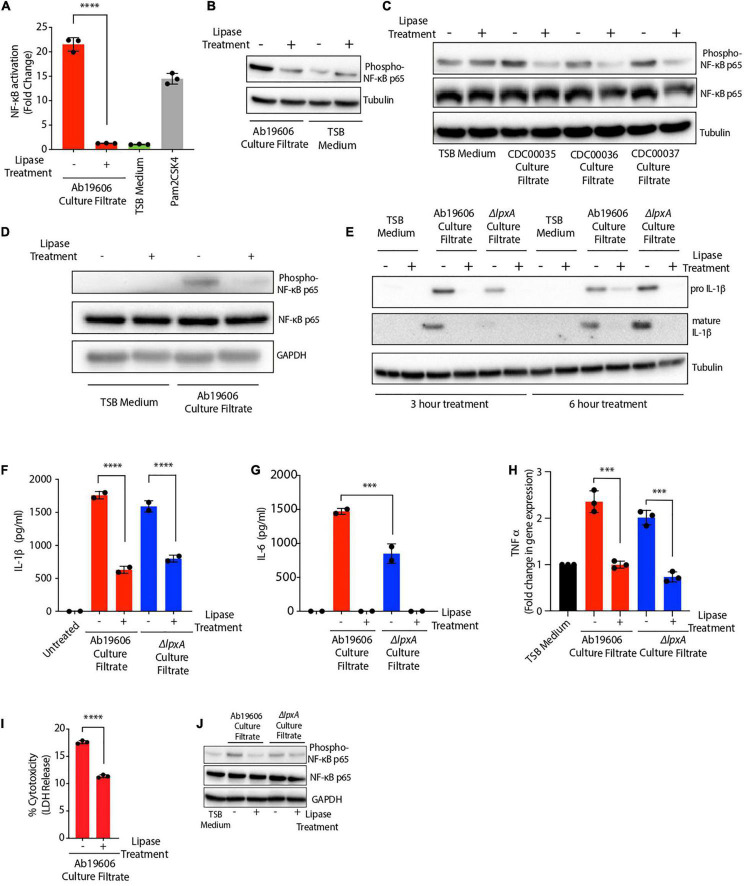
*Acinetobacter baumannii* secretes a bioactive lipid that triggers immune signaling. **(A)** Culture filtrate from wildtype *A. baumannii* (Ab19606) was treated with lipase enzyme overnight. THP1-XBlue reporter cells were exposed to untreated and the lipase treated culture filtrate. Levels of SEAP were assessed after 24 h of culture filtrate treatment. The experiments were done in triplicates. TSB medium was used to grow the bacterial cultures and fresh TSB medium treatment served as the negative control while Pam2CSK4 served as the positive control. Error bars represent standard deviation. One-way ANOVA with Tukey’s multiple comparisons test. **(B–D)** THP1 macrophages **(B,C)** and RAW264.7 macrophages **(D)** were exposed to untreated or lipase treated culture filtrates from the indicated bacteria. Western blot was performed to check the phosphorylation of NF-κB p65. **(E)** THP1 macrophages were exposed to untreated or lipase treated culture filtrates from the indicated bacteria for 3 and 6 h. Western blot was performed to check the levels of pro and mature forms of IL-1ß protein. **(F–H)** THP1 macrophages were exposed to untreated or lipase treated culture filtrate. Levels of secreted IL-1ß **(F)**, and IL-6 **(G)** were assessed after 24 h of culture filtrate treatment and gene expression of TNFα **(H)** was assessed after 3 h of culture filtrate treatment. **(I)** THP1 macrophages were exposed to untreated or lipase treated culture filtrate. LDH levels in the cell culture supernatant were assessed after 24 h of culture filtrate treatment. The experiments were done in triplicates. Error bars represent standard deviation. **(J)** A549 cells were exposed to untreated or lipase treated culture filtrates from the indicated bacteria. Western blot was performed to check the phosphorylation of NF-κB p65. One-way ANOVA with Tukey’s multiple comparisons test. All the experiments were performed three times independently. *P < 0.05, **P < 0.01, ***P < 0.001, ****P < 0.0001.

Given the hydrophobic nature of the bioactive inflammatory factor, we hypothesized that the activating factor might be a lipid. To test this hypothesis, we treated *A. baumannii* culture filtrate with lipase to degrade all the lipids. Indeed, lipase treatment of the culture filtrate completely abolished NF-κB activation (Figure 4A). These results were further confirmed by western blotting in THP1 macrophages where NF-κB phosphorylation was not observed upon exposure of cells to lipase treated culture filtrate (Figure 4B). Lipase treatment of culture filtrates from *A. baumannii* clinical isolates also abrogated their ability to activate NF-κB in THP1 macrophages suggesting the secretion of the inflammatory bioactive lipid by *A. baumannii* clinical isolates (Figure 4C). Lipase treated *A. baumannii* culture filtrate also lost the ability to activate NF-κB in murine RAW264.7 macrophages (Figure 4D). *A. baumannii* culture filtrate also induced the expression of pro and mature forms of IL-1ß protein in THP1 macrophages which was not observed in macrophages exposed to lipase-treated culture filtrate (Figure 4E). Wildtype *A. baumannii* culture filtrate treatment displayed early induction of mature IL-1ß protein (3 h post-treatment) while culture filtrate from Δ*lpxA* displayed a delayed response in the induction of mature IL-1ß protein (6 h post-treatment) (Figure 4E). Consistent with the western blot results, treatment of THP1 macrophages with lipase-treated *A. baumannii* culture filtrate significantly reduced the secretion of IL-1ß (Figure 4F). Moreover, lipase treatment of *A. baumannii* culture filtrate also abolished its ability to induce IL-6 secretion in THP1 macrophages (Figure 4G). Tumor necrosis factor (TNF) α, which is another important cytokine downstream of NF-κB, was also transcriptionally induced in THP1 macrophages treated with *A. baumannii* culture filtrate. However, TNFα induction was also dependent on the presence of the bioactive lipid in the bacterial culture filtrate (Figure 4H). We also monitored cell death and observed a partial but significant reduction in cell death when THP1 macrophages were exposed to *A. baumannii* culture filtrate previously treated with the lipase enzyme (Figure 4I). We further confirmed the effects of *A. baumannii* culture filtrate in epithelial cells. A549 human lung epithelial cells treated with the culture filtrate from both wildtype and Δ*lpxA A. baumannii* exhibited NF-κB phosphorylation. However, NF-κB phosphorylation was reduced in cells exposed to bacterial culture filtrate previously treated with lipase suggesting that the bioactive lipid triggers NF-κB activation in epithelial cells also (Figure 4J). Collectively, these results suggest that *A. baumannii* secretes a bioactive lipid which activates the NF-κB and the inflammasome signaling pathways, leading to IL-1ß, IL-6 and TNFα secretion and cell death.

### *Acinetobacter baumannii* Secretes a TLR2 Activating Lipid That Triggers Canonical NF-κB Signaling

**FIGURE 5:**
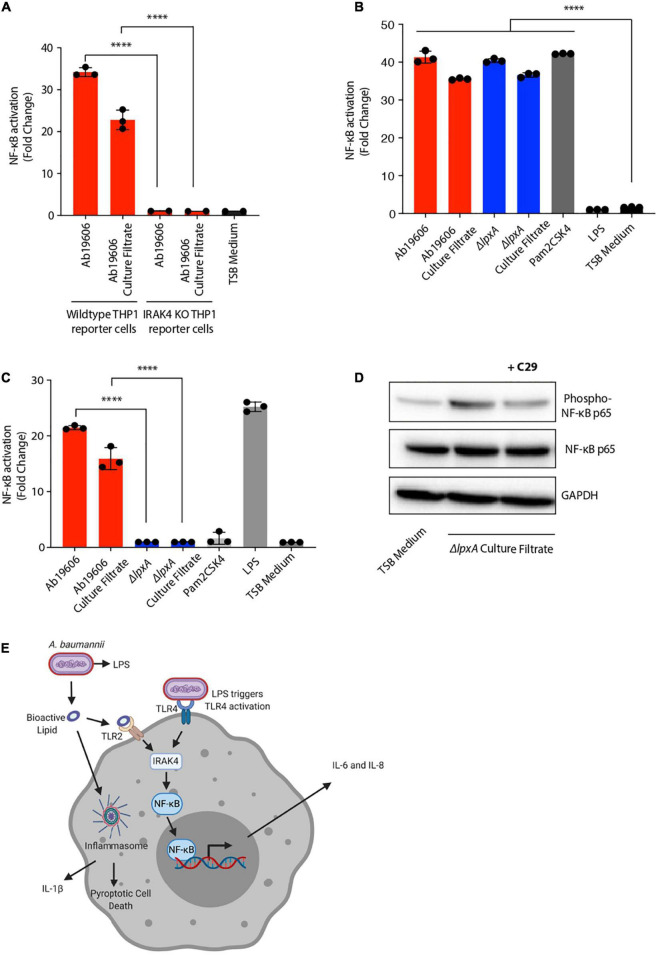
*Acinetobacter baumannii* secretes a TLR2 activating lipid that triggers canonical NF-κB signaling. **(A)** Wildtype THP1-XBlue reporter cells or THP1-XBlue *IRAK4^–/–^* reporter cells were infected with wildtype *A. baumannii* (Ab19606) or treated its culture filtrate. TSB medium was used to grow the bacterial cultures and fresh TSB medium treatment served as the negative control. Levels of SEAP were assessed after 24 h from the beginning of the treatment. The experiments were done in triplicates. Error bars represent standard deviation. One-way ANOVA with Tukey’s multiple comparisons test. **(B,C)** HEK293 reporter cells stably expressing TLR2 **(B)** or TLR4 **(C)** were infected with wildtype *A. baumannii* (Ab19606) or Δ*lpxA* at MOI 10 or were treated with the respective culture filtrates. Levels of SEAP were assessed after 24 h of infection or culture filtrate treatment. TSB medium was used to grow the bacterial cultures and fresh TSB medium treatment served as the negative control. Pam2CSK4 and LPS served as additional controls. The experiments were done in triplicates. Error bars represent standard deviation. One-way ANOVA with Tukey’s multiple comparisons test. **(D)** THP1 macrophages treated with or without the TLR2 inhibitor C29 were exposed to Δ*lpxA* culture filtrate. Western blot was performed to check the phosphorylation of NF-κB p65. **(E)** Schematic representation depicting the secretion of the bioactive lipid by *A. baumannii* that activates inflammatory signaling (created with BioRender.com). All the experiments were performed three times independently. *P < 0.05, **P < 0.01, ***P < 0.001, ****P < 0.0001.

NF-κB signaling is known to have two distinct paths of activation: the canonical and the non-canonical pathways, each of which is activated by distinct stimuli and regulate downstream immune responses (Yu et al., 2020). IRAK4 is a critical member of the IL-1 receptor-associated kinase (IRAK) family of proteins which is required for NF-κB activation in the canonical signaling pathway (Kawai and Akira, 2007; Kawasaki and Kawai, 2014; Yu et al., 2020). Infection of *IRAK4*^–/–^ reporter cells with *A. baumannii* or bacteria-free culture filtrate treatment resulted in complete loss of NF-κB activation suggesting that IRAK signaling is required for the activation of NF-κB during *A. baumannii* infection and culture filtrate treatment (Figure 5A). These results suggest that the novel secreted bioactive lipid induces canonical NF-κB signaling.

Having established the involvement of IRAK signaling during *A. baumannii* infection and culture filtrate treatment, we next sought to determine the host receptor that is required for detecting the bioactive lipid in *A. baumannii* culture filtrate. TLR signaling is the major contributor to IRAK dependent NF-κB activation and TLR signaling is known to be activated upon *A. baumannii* infection (Chen, 2020). In order to identify the host receptor responsible for the activation NF-κB upon treatment with *A. baumannii* culture filtrate, we used the human embryonic kidney (HEK) 293 cells stably expressing either TLR2 or TLR4 and an NF-κB inducible SEAP reporter gene. This is a widely used reporter system to study receptor activation (Li et al., 2020). Pam2CSK4, which is a TLR2 agonist, and LPS, which is a TLR4 agonist, were used to validate and verify the activity of the reporter cell assay (Figures 5B,C). Upon treatment of TLR2-expressing reporter cells with wildtype *A. baumannii* and Δ*lpxA* culture filtrates, significant NF-κB activation was observed (Figure 5B). While the culture filtrate from wildtype *A. baumannii* also induced NF-κB in TLR4 expressing reporter cells, the activation was completely abolished in TLR4-expressing reporter cells when treated with Δ*lpxA* culture filtrate (Figure 5C). This indicates that TLR2 acts as the receptor to sense the bioactive lipid in *A. baumannii* culture filtrate that further leads to NF-κB activation (Figure 5C). To further validate this finding in macrophages, we used an inhibitor (C29) to block the activity of TLR2. C29 treatment reduced NF-κB activation upon Δ*lpxA* culture filtrate treatment in THP1 macrophages (Figure 5D). Taken together these data suggest that *A. baumannii* secretes an inflammatory bioactive lipid that activates TLR2 to stimulate downstream inflammatory signaling via canonical NF-κB activation (Figure 5E).

## Discussion

Innate immune signaling is the first line of defense that activates downstream pro-inflammatory cytokines to tackle infections. To do this efficiently, host cells employ a plethora of different PRRs that can sense infections and insults by recognizing a variety of PAMPs (Thaiss et al., 2016; Tiku et al., 2020). One of the most well studied PAMPs is LPS which is abundantly present in the outer membrane of Gram-negative bacteria and is detected by the host receptor TLR4. Directly shed LPS or LPS in association with OMVs, activates different nodes of immune signaling including TLR4 driven NF-κB activation and caspase 11 driven inflammasome activation (Lin et al., 2012; Vanaja et al., 2016). Upon *A. baumannii* infections, LPS is known to activate the TLR4 signaling, however, it is not known if *A. baumannii* secretes any immunomodulatory factors that can alarm the immune system (Lin et al., 2012). *A. baumannii* expresses multiple secretion systems, yet very little is known about the toxins and other potentially pathogenic and inflammatory factors that it secretes to enhance its virulence and evade host immunity (Harding et al., 2017; Chen, 2020). Release of OMVs has been reported to be a virulence factor secretion strategy employed by *A. baumannii*, nevertheless other pathogenic secretions of *A. baumannii* remain broadly unexplored (Jin et al., 2011; Tiku et al., 2021).

In this study, we report *A. baumannii* secretes a bioactive lipid which signals via TLR2 to activate canonical NF-κB signaling in human and murine macrophages in an IRAK4-dependent manner leading eventually to the production of pro-inflammatory cytokines IL-6, IL-8 and TNFα. The inflammatory activity was not limited to only NF-κB signaling, but it also led to the activation of the inflammasome pathway and the subsequent induction of cell death (Figure 5E). Interestingly culture filtrates from an LPS-deficient mutant Δ*lpxA* and lipoprotein mutants are still able to activate NF-κB signaling which suggests the presence of a novel inflammatory lipid factor. Treating the culture filtrate with lipase to hydrolyze lipids was the only treatment that completely abrogated NF-κB activation, IL-6 secretion and partially abolished IL-1ß secretion and cell death demonstrating that the activating signal is a bioactive lipid. Our findings also suggest that the inflammatory lipid factor induces inflammatory signaling in both macrophages and epithelial cells highlighting shared mechanisms of induction of immune signaling. Major bacterial lipids that are known to induce inflammatory responses are broadly membrane associated such as LPS in Gram-negative bacteria and lipoteichoic acid (LTA) in Gram-positive bacteria (Ernst and Chandler, 2017). Our biochemical and cell-based assay data suggest the presence of a secreted bioactive lipid from *A. baumannii* which can induce the activation of inflammatory cytokines and cell death.

In order to identify the host cell receptor that detects the specific inflammatory lipid species under investigation, we used HEK293 reporter cells stably expressing either TLR2 or TLR4. *A. baumannii* culture filtrate activated NF-κB both via TLR2 and TLR4 signaling. TLR4 expressing cells when treated with the culture filtrate from Δ*lpxA* mutant did not show activation of NF-κB while in TLR2 expressing cells NF-κB activation was observed with the culture filtrates from both the wildtype *A. baumannii* and Δ*lpxA* mutant. Furthermore, blocking TLR2 signaling in macrophages reduced NF-κB activation upon treatment with *A. baumannii* culture filtrate confirming the role of TLR2 signaling in immune activation upon *A. baumannii* culture filtrate exposure. These results establish the presence of an immune-stimulatory lipid species secreted by *A. baumannii* that signals through a mechanism distinct from LPS. While it is known that upon *A. baumannii* infection both TLR2 and TLR4 are activated it is unclear if the ligands are secreted or if the activation is because of a direct interaction of bacteria with the host cells (Knapp et al., 2006). Our work is suggestive of the presence of a secreted lipid species that leads to TLR2 stimulation and downstream NF-κB activation. The effects of TLR4 are well established for *A. baumannii* infection where loss of TLR4 leads to increased bacterial burden in a mouse lung infection model (Lin et al., 2012). However, there are conflicting reports about the role of TLR2 in *A. baumannii* infection. One study reported higher bacterial burden in the lungs of TLR2 knock out mice while another study reported a reduction in bacterial numbers (Knapp et al., 2006; Kim et al., 2014). Even though the exact role of TLR2 in the context of bacterial clearance in mouse infection models of *A. baumannii* remains to be resolved, our study reveals the activation of the TLR2 signaling pathway with an *A. baumannii* secreted lipid. *A. baumannii* has a peculiar lipid profile. It possesses unique glycerophospholipds especially high levels of monolysocardiolipin which is rarely detected in bacteria (Lopalco et al., 2017). Cardiolipin can activate both the NF-κB signaling via TLR2 and the NLRP3 inflammasome pathway (Cho et al., 2018; Pizzuto and Pelegrin, 2020). Since we observe activation of both these pathways, it is plausible that *A. baumannii* secretes monolysocardiolipin which triggers the PRRs to mount an inflammatory response. Future studies will focus on unraveling the role of this bioactive lipid *in vivo* to examine if it induces inflammation in mice and if pre-treatment of mice with *A. baumannii* culture filtrate induces immunity against a subsequent *A. baumannii* infection.

## Materials and Methods

### Bacterial Strains and Bacterial Culture Conditions

*Acinetobacter baumannii* bacterial strains used in this study along with their sources are as follows: Ab19606 and Ab17978 were obtained from the American Type Culture Collection (ATCC). The multidrug resistant clinical isolates Ab5075, CDC35, CDC36, CDC37 were obtained from the University of Washington (Seattle, WA) and The Centers for Disease Control and Prevention (CDC). Lipoprotein mutants (in Ab5075 genetic background) Δ*lspA*, Δ*lnt* and Δ*lgt* were obtained from the *A. baumannii* transposon mutant library (Gallagher et al., 2015). Bacteria were streaked out on tryptic soy agar (TSA) plates from frozen glycerol stocks and incubated at 37°C overnight. Single colonies from these overnight plates were used to inoculate liquid cultures using tryptic soy broth (TSB) medium. All the bacterial strains used in this study are listed in Supplementary Table 2.

### Bacterial Genetic Recombineering

Previously described genetic recombineering in *A. baumannii* was followed to generate Δ*lpx*A mutant lacking LPS (Tucker et al., 2014; Bojkovic et al., 2016). A kanamycin cassette containing 500 base pairs of *lpxA* flanking sequences was synthesized. 5 μg of the construct DNA was electroporated into competent *A. baumannii* expressing Rec_AB_ recombinase previously induced by 2 mM isopropyl ß-D-1-thiogalactopyranoside (IPTG). After electroporation the bacteria were recovered in 4 ml TSB medium with 2 mM IPTG for 4 h. The bacterial culture was centrifuged and the supernatant was removed and the final leftover volume containing the bacteria was streaked out on TSB plates containing 50 μg/ml kanamycin. Multiple colonies were examined by PCR for the absence of the *lpxA* gene and the presence of the kanamycin cassette instead. Mutants were verified by PCR and DNA sequencing for the deletion of *lpxA. ΔompA* strain was also generated using the same protocol as also detailed in our previous study (Tiku et al., 2021).

### Cell Culture

Mammalian cells used in this study included RAW264.7, THP1, HEK293 (HEK-Blue TLR2/4 reporter cells – InvivoGen), THP1-XBlue reporter cells (InvivoGen) and A549. All the cells were cultured in Roswell Park Memorial Institute Medium (RPMI) supplemented with 10% v/v fetal bovine serum (FBS), 2 mM glutamine and 10 mM HEPES buffer. For maintenance the cells were passage two to three times per week.

### *In vitro* Infections and Culture Filtrate Treatment

For infections and culture filtrate treatments of mammalian cells, 10^5^ cells per well were seeded in a 96-well plate a day before the experiment. In parallel, bacterial cultures were grown overnight in TSB medium. The next day, bacterial cultures were back-diluted to grow fresh exponential phase cultures for infection. The overnight cultures were centrifuged at 4500 rpm to pellet bacteria and the supernatant was filtered using 0.2 μm filters to remove any bacteria. Complete removal of bacteria from the culture filtrate was further verified by plating of culture filtrate on TSB plates. Mammalian cells were washed with PBS once and infected at MOI of 10 for 2 h after which 200μg/ml gentamicin was added to kill extracellular bacteria. For the cells that were treated with the culture filtrate, the treatment was continued overnight. The next day supernatant from the cells infected with bacteria and treated with the culture filtrate was used to assess the levels of secreted SEAP using the QUANTI-Blue kit (Invivogen) following the manufacturer’s protocol.

### Western Blotting

Standard western blotting protocol was followed as previously described (Tiku et al., 2018). Briefly western blotting samples were prepared lysing 10^6^ cells/well in 150 μl of RIPA buffer containing protease inhibitors. The cell lysis was continued at 4°C for half an hour followed by protein estimation using a bicinchoninic acid (BCA) assay kit (ThermoFisher Scientific) was performed. Sodium dodecyl sulfate polyacrylamide gel electrophoresis (SDS-PAGE) was further performed by loading 20 μg of protein for each sample. The gel was blotted onto a nitrocellulose membrane and blocked for an hour in the blocking buffer at room temperature. Primary antibodies were used at 1:1000 dilution and incubated overnight. The next day after three washes in tris-buffered saline with 0.1% tween 20 detergent (TBST), secondary antibodies were added at 1:10,000 dilution followed by an hour of incubation at room temperature after which the membranes were developed and imaged. The following antibodies were used: Phospho-NF-κB p65 (Ser536) (Cell Signaling Technology 3033S) and NF-κB p65 (Cell Signaling Technology 6956S).

### Biochemical Fractionation

Fractionation of the culture filtrate was adapted from a previously published study (Woodward et al., 2010). From an overnight *A. baumannii* culture, a fresh 50 ml culture was inoculated in TSB medium and grown for 2 h at 37°C. The culture was then centrifuged and the pelleted bacteria were resuspended in PBS containing 0.2% glucose. The culture was further grown overnight at 37°C. The next day the culture was centrifuged and the supernatant was filtered through a 0.2μm filter. pH of this culture filtrate was adjusted to 4. Sep-Pak C18 columns (Waters Corporation) were used for fractionating the culture filtrate. 15 ml of the culture filtrate was then run through the column and the flow through was collected. After the run, the column was eluted sequentially with 0.1% trichloroacetic acid (TCA) in water, 0.1% TCA+50% methanol (at a 1:1 ratio) and 100% methanol. Eluate was sequentially collected as 1 ml fractions and all the collected fractions were vacuum dried and the left-over pellet was resuspended in 500 μl of sterile water. 50 μl from each fraction was tested for its ability to activate NF-κB using the THP1-XBlue reporter cells.

### Organic Extraction

Culture filtrate from the overnight grown wildtype *A. baumannii* was mixed chloroform at a 1:1 ratio. The samples were vortexed vigorously followed by centrifugation at 4000 rpm for 10 min to separate out the organic and the aqueous phases. After centrifugation, the phases were separately collected. The top phase was the aqueous phase while the bottom one was the organic phase. Both phases collected separately were dried and the dried-out pellets were resuspended in 200 μl of sterile water. THP1-XBlue reporter cells were treated with the resuspended pellets from the two phases for 24 h. The supernatant from the treated reporter cells was used to assess the levels of secreted SEAP using the QUANTI-Blue kit (Invivogen) following the manufacturer’s protocol.

### Enzyme-Linked Immunosorbent Assay

Levels of cytokines IL-6, IL-8 and IL-1ß were examined by commercially available ELISA kits from ThermoFisher Scientific (Catalog numbers EH2IL6, KHC0081 and BMS224-2). THP1 monocytes differentiated into macrophages with 80 ng/ml phorbol 12-myristate 13-acetate (PMA) for 48 h were infected with wildtype or Δ*lpxA* mutant or treated with the culture filtrates from these bacteria for 24 h. Cell culture supernatants were collected 24 h post-infection or culture filtrate treatment and cytokine levels were assayed by using the ELISA kits following manufacturer’s protocol.

### Immunofluorescence

3.2 x10^5^ THP1 monocytes/chamber were seeded in a 4-chamber slide. 80 ng/ml PMA for 48 h was used to differentiate the cells into macrophages. After differentiation bacterial cultural filtrate treatment was performed for 6 h after which the cells were washed twice with PBS and fixed with 4% paraformaldehyde (PFA) and then permeabilized with PBS containing 0.1% triton-X 100 for 15 min at room temperature. The cells were then blocked with 1% bovine serum albumin (BSA) for an hour at room temperature after which primary antibody treatment (anti-ASC antibody from Novus Biologicals, diluted in BSA at 1:200) was performed. After 1 h of incubation with the primary antibody at room temperature, the cells were washed three times with PBS and treated with fluorescently labeled (Alexa594) secondary antibody for 1-h room temperature. The cells were then mounted with ProLong Gold mounting medium containing DAPI. Confocal imaging was performed with Nikon Ti-E spinning disk confocal microscope. All the images were acquired using the 100X and 60X oil immersion objectives. Image analysis and processing was performed using Fiji open-source software.

## Data Availability Statement

The original contributions presented in the study are included in the article/Supplementary Material, further inquiries can be directed to the corresponding authors.

## Author Contributions

VT, M-WT, and ID conceptualized the study. VT and CK performed the experimental work. VT performed data analysis. EK helped with designing and setting up experiments. YP generated the Δ*lpxA* mutant strain. VT and M-WT wrote the manuscript. All authors contributed to the article and approved the submitted version.

## Conflict of Interest

VT, EK, YP and M-WT were employed by company Genentech. VT is currently employed by Vir Biotechnology. The remaining authors declare that the research was conducted in the absence of any commercial or financial relationships that could be construed as a potential conflict of interest. The authors declare that this study received funding from Genentech. The funder was not involved in the study design, collection, analysis, interpretation of data, the writing of this article or the decision to submit it for publication.

## Publisher’s Note

All claims expressed in this article are solely those of the authors and do not necessarily represent those of their affiliated organizations, or those of the publisher, the editors and the reviewers. Any product that may be evaluated in this article, or claim that may be made by its manufacturer, is not guaranteed or endorsed by the publisher.

## Supplementary Material

Supplementary Figure 1 **(A)** Overnight grown wildtype *A. baumannii* (Ab19606) and its culture filtrate were streaked out on TSB plates to assess bacterial numbers (colony forming units or CFUs). **(B)** Schematic representation of genetic recombineering to generate Δ*lpxA* strain (created with BioRender.com). **(C,D)** THP1-XBlue reporter cells treated with culture filtrates from the indicated bacteria. TSB medium was used to grow the bacterial cultures and fresh TSB medium treatment served as the negative control. Levels of SEAP were assessed after 24 h from the beginning of the treatment. The experiments were done in triplicates. Error bars represent standard deviation. One-way ANOVA with Tukey’s multiple comparisons test ^****^*p* 0.0001.

Supplementary Figure 2 **(A)** Culture filtrate from wildtype *A. baumannii* (Ab19606) was treated with phosphodiesterase (PDE). THP1-XBlue reporter cells were exposed to untreated and the treated culture filtrate. Levels of SEAP were assessed after 24 h of culture filtrate treatment. The experiments were done in triplicates. Error bars represent standard deviation. One-way ANOVA with Tukey’s multiple comparisons test ^****^*p* 0.0001. **(B)** Efficiency of the phosphodiesterase (PDE) enzyme was verified in samples containing 5μM ATP with or without PDE treatment by using BacTiter-Glo assay. **(C)** THP1-XBlue reporter cells were treated with the indicated culture filtrates with or without OMVs. TSB medium was used to grow the bacterial cultures and fresh TSB medium treatment served as the negative control. Levels of SEAP were assessed after 24 h from the beginning of the treatment. The experiments were done in triplicates. Error bars represent standard deviation. One-way ANOVA with Tukey’s multiple comparisons test ^****^*p* 0.0001. **(D)** THP1-XBlue reporter cells were treated with the indicated culture filtrates. TSB medium was used to grow the bacterial cultures and fresh TSB medium treatment served as the negative control. Levels of SEAP were assessed after 24 h from the beginning of the treatment. The experiments were done in triplicates. Error bars represent standard deviation. One-way ANOVA with Tukey’s multiple comparisons test ^****^*p* 0.0001.
