# Supplementary figures and images for: Acinetobacter baumannii Secretes a Bioactive Lipid That Triggers Inflammatory Signaling and Cell Death
Source: Front Microbiol. 2022 May 9;13:870101. doi: 10.3389/fmicb.2022.870101 (PMC9125205; doi:10.3389/fmicb.2022.870101)

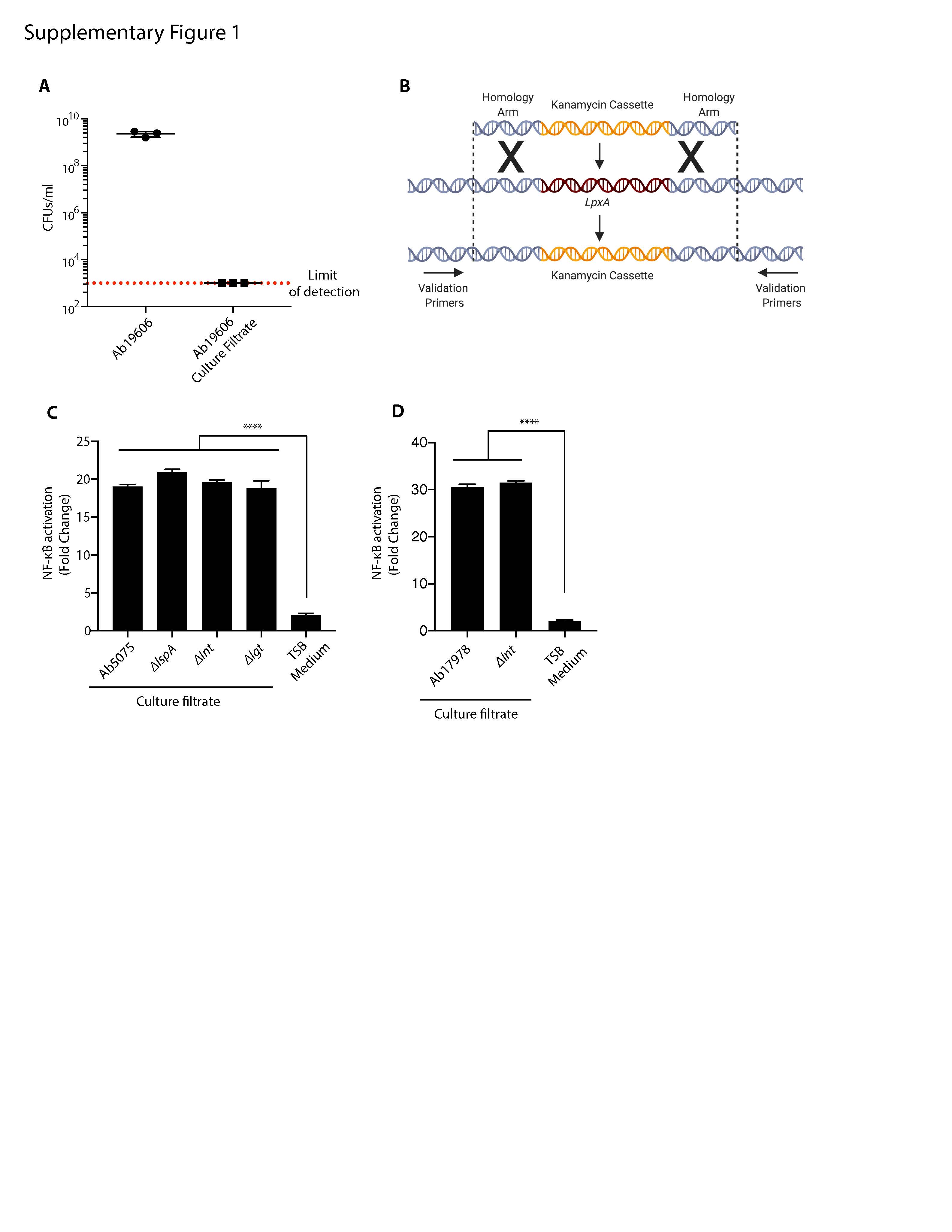

Supplement: Supplementary file 1 [file Image_1.JPEG]

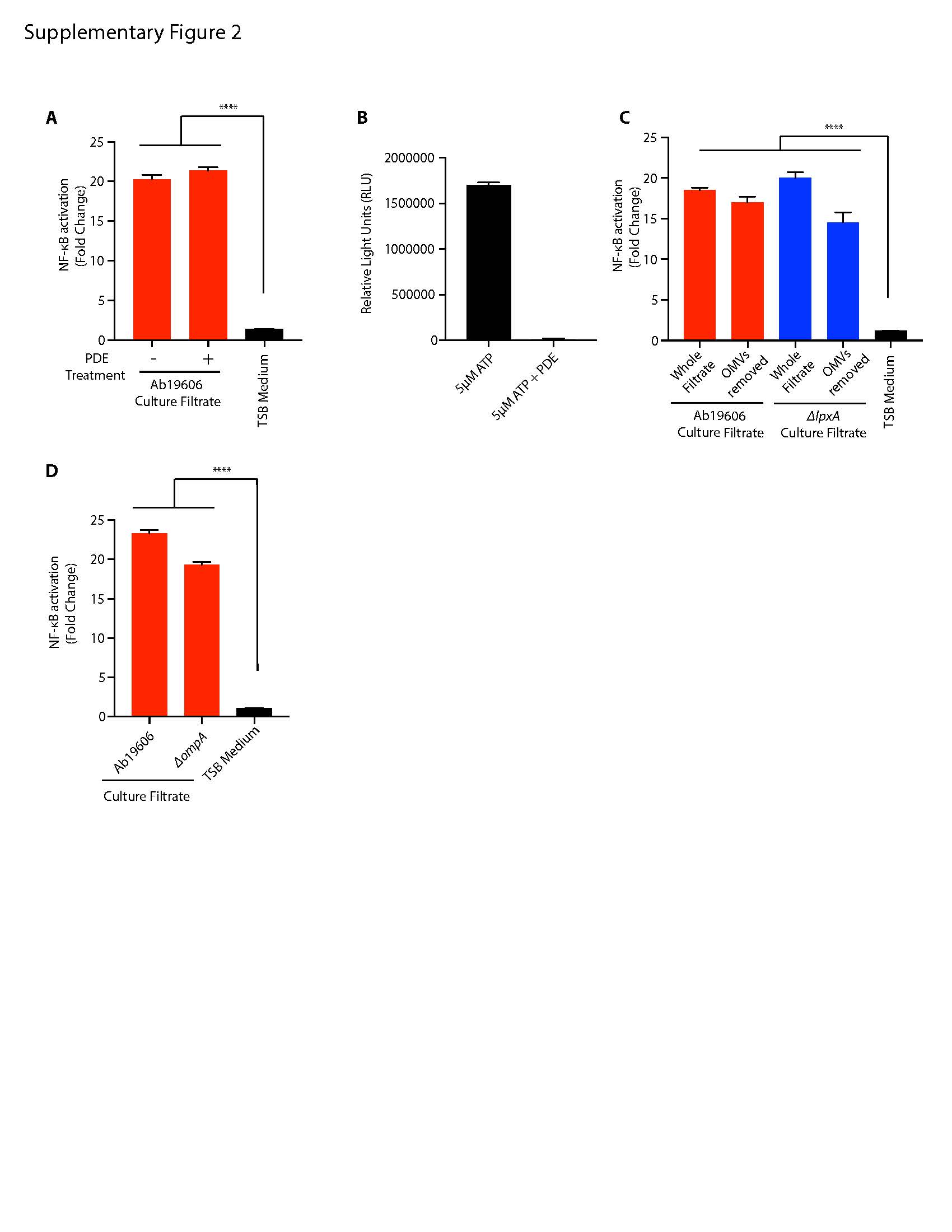

Supplement: Supplementary Figure 1 — (A) Overnight grown wildtype A. baumannii (Ab19606) and its culture filtrate were streaked out on TSB plates to assess bacterial numbers (colony forming units or CFUs). (B) Schematic representation of genetic recombineering to generate ΔlpxA strain (created with BioRender.com). (C,D) THP1-XBlue reporter cells treated with culture filtrates from the indicated bacteria. TSB medium was used to grow the bacterial cultures and fresh TSB medium treatment served as the negative control. Levels of SEAP were assessed after 24 h from the beginning of the treatment. The experiments were done in triplicates. Error bars represent standard deviation. One-way ANOVA with Tukey’s multiple comparisons test ****p 0.0001. [file Image_2.JPEG]

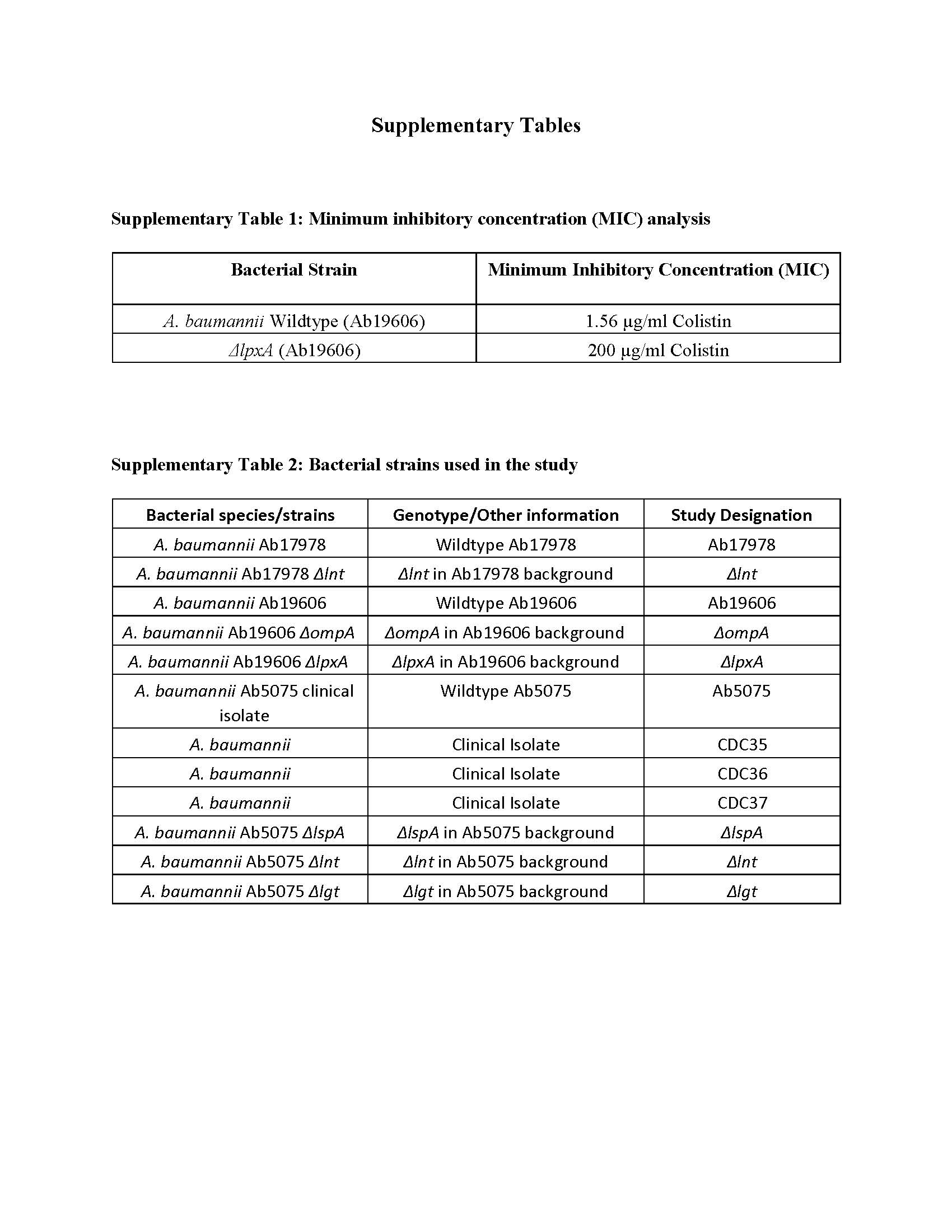

Supplement: Supplementary Figure 2 — (A) Culture filtrate from wildtype A. baumannii (Ab19606) was treated with phosphodiesterase (PDE). THP1-XBlue reporter cells were exposed to untreated and the treated culture filtrate. Levels of SEAP were assessed after 24 h of culture filtrate treatment. The experiments were done in triplicates. Error bars represent standard deviation. One-way ANOVA with Tukey’s multiple comparisons test ****p 0.0001. (B) Efficiency of the phosphodiesterase (PDE) enzyme was verified in samples containing 5μM ATP with or without PDE treatment by using BacTiter-Glo assay. (C) THP1-XBlue reporter cells were treated with the indicated culture filtrates with or without OMVs. TSB medium was used to grow the bacterial cultures and fresh TSB medium treatment served as the negative control. Levels of SEAP were assessed after 24 h from the beginning of the treatment. The experiments were done in triplicates. Error bars represent standard deviation. One-way ANOVA with Tukey’s multiple comparisons test ****p 0.0001. (D) THP1-XBlue reporter cells were treated with the indicated culture filtrates. TSB medium was used to grow the bacterial cultures and fresh TSB medium treatment served as the negative control. Levels of SEAP were assessed after 24 h from the beginning of the treatment. The experiments were done in triplicates. Error bars represent standard deviation. One-way ANOVA with Tukey’s multiple comparisons test ****p 0.0001. [file Image_3.JPEG]
